# Supplementary material for: Associations between vitamin D and autoimmune diseases: Mendelian randomization analysis
Source: Semin Arthritis Rheum. Author manuscript; Available in PMC 2023 Jul 21. (PMC7614794; doi:10.1016/j.semarthrit.2023.152238)
Supplement: Supplementary Material [file EMS180734-supplement-Supplementary_Material.docx]

**Supplementary materials**

**Associations between vitamin D and autoimmune diseases: Mendelian randomization analysis**

Sizheng Steven Zhao MD PhD^1^, Amy Mason PhD^2,3,4^, Eva Gjekmarkaj^4^,

1 Centre for Epidemiology Versus Arthritis, Division of Musculoskeletal and Dermatological Science, School of Biological Sciences, Faculty of Biological Medicine and Health, The University of Manchester, Manchester Academic Health Science Centre, Manchester, UK

2 British Heart Foundation Cardiovascular Epidemiology Unit, Department of Public Health and Primary Care, University of Cambridge, Cambridge, UK

3 Heart and Lung Research Institute, University of Cambridge, Cambridge UK

4 Medical Research Council Biostatistics Unit, University of Cambridge, Cambridge, UK

5 Department of Allergy and Rheumatology, Graduate School of Medicine, The University of Tokyo, Tokyo, Japan.

Contents

[Supplementary Methods 2](#_Toc137560449)

[Supplementary Table 1. Autoimmune disease and control outcome definitions 2](#_Toc137560450)

[Supplementary Table 2. Mean values of 25(OH)D concentration (nmol/L) by month of blood draw 4](#_Toc137560451)

[Supplementary Table 3. List of genetic variants in the genetic risk score 4](#_Toc137560452)

[Supplementary Table 4. Non-linear associations between 25(OHD) and each outcome across five quantiles. 5](#_Toc137560453)

[Supplementary Table 5. Two-sample MR pleiotropy robust sensitivity analyses. 9](#_Toc137560454)

# Supplementary Methods

In each forward step, genetic variants were ranked based on their associations with 25(OH)D concentrations conditional on variants selected at any previous step. At each step, the variant having the lowest p-value was selected. The process was repeated for each locus until no further variants were conditionally associated with 25(OH)D concentrations at a genome-wide level of significance (p < 5×10-8). Finally, a backward step was applied to omit any variant failing to meet the genome-wide level of significance for association with 25(OH)D concentrations in a joint model including all selected variants [1]. The motivation of this approach is to select variants that explain independent variability in the exposure (even if they may be in partial linkage disequilibrium); increasing the variance explained in the exposure by the genetic variants increases the power of the Mendelian randomization analysis.

1. Sofianopoulou E, Kaptoge SK, Afzal S, Jiang T, Gill D, Gundersen TE, et al. Estimating dose-response relationships for vitamin D with coronary heart disease, stroke, and all-cause mortality: observational and Mendelian randomisation analyses. The Lancet Diabetes & Endocrinology 2021;9:837–46.

# Supplementary Table 1. Autoimmune disease and control outcome definitions

| **Outcome name** | **ICD-9 codes** | **ICD-10 codes** | **Self-report** | **N** |
| --- | --- | --- | --- | --- |
| Rheumatoid arthritis | 714.0, 714.1, 714.2, 714.81 | M05.0, M05.1, M05.2, M05.3, M05.8, M05.9, M06.0, M06.8, M06.9 | 1464 | 5172 |
| Systemic lupus erythematosus | 710.0 | M32.0, M32.1, M32.8, M32.9 | 1424 | 527 |
| Systemic sclerosis | 710.1 | M34.0, M34.1, M34.2, M34.8, M34.9 | 1384 | 201 |
| Sjogren’s syndrome | 710.2 | M35.0 | 1382 | 335 |
| Graves’ disease | 242.0 | E05.X | 1522 | 1798 |
| Hashimoto’s thyroiditis | 245.2 | E06.3 | - | 194 |
| Coeliac disease | 579.0 | K90.0 | 1456 | 2249 |
| Type 1 diabetes mellitus | 250.01 | E10 | 1222 | 341 |
| Primary biliary cholangitis | 571.6 | K74.3 | 1506 | 243 |
| Autoimmune hepatitis | 571.42 | K75.4 | - | 153 |
| Polymyalgia rheumatica | 725 | M35.3 | 1377 | 1780 |
| Giant cell arteritis | 446.5 | M31.5, M31.6 | 1376 | 436 |
| Polyarteritis nodosa | 446.0 | M30.0 | 1380 | 91 |
| Henoch-Schönlein Purpura | 287.0 | D69.0 | - | 47 |
| Granulomatosis with polyangiitis | 446.4 | M31.3 | 1378 | 62 |
| Eosinophilic granulomatosis with polyangiitis | - | M30.1 | - | 46 |
| Microscopic polyangiitis | - | M31.7 | 1379 | 19 |
| Mixed connective tissue disease | - | M35.1 | - | 41 |
| Antiphospholipid syndrome | - | D68.6 | 1564 | 28 |
| Dermato/polymyositis | 710.3, 710.4 | M33.1, M33.2, M33.9 | 1383 | 6 |
| Ankylosing spondylitis | 720.0 | M45 | 1313 | 1121 |
| Psoriatic arthritis | 696.0 | M07.0, M07.2, M07.3 | 1477 | 703 |
| Psoriasis | 696.1 | L40.0, L40.1, L40.2, L40.3, L40.4, L40.8, L40.9 | 1453 | 5120 |
| Crohn’s disease | 555.X | K50.X | 1462 | 1795 |
| Ulcerative colitis | 556.X | K51.X | 1463 | 3461 |
| Primary sclerosing cholangitis | 714.0, 714.1, 714.2, 714.81 | M05.0, M05.1, M05.2, M05.3, M05.8, M05.9, M06.0, M06.8, M06.9 | 1464 | 35 |
| Behcet's disease | 136.1 | M35.2 | - | 30 |
| Takayasu arteritis | 446.7 | M31.4 | - | 6 |
| Kawasaki disease | 446.1 | M30.3 | - | 1 |
| Multiple sclerosis | 340 | G35 | 1261 | 1504 |
| Osteoarthritis | 715.X | M15.0, M15.1, M15.2, M15.3, M15.4, M15.8, M15.9, M19.9 | 1465 | 31628 |

International Classification of Diseases (ICD) codes were used to match against conditions and cause of death, self-report codes were used to match against illness codes in UK Biobank data field 20002 (“non-cancer illness code, self-reported”). The .X notation means that any subcode is matched. Only diseases with n>100 were included in analyses.

# Supplementary Table 2. Mean values of 25(OH)D concentration (nmol/L) by month of blood draw

| **Month** | **Mean 25(OH)D concentration (nmol/L)** |
| --- | --- |
| January | 44.4 |
| February | 40.1 |
| March | 39.4 |
| April | 39.5 |
| May | 42.0 |
| June | 47.0 |
| July | 55.1 |
| August | 59.7 |
| September | 60.7 |
| October | 60.0 |
| November | 55.4 |
| December | 48.5 |

# Supplementary Table 3. List of genetic variants in the genetic risk score

| Chromosome: Position (hg19) | rsID | Effect allele | Other allele | Minor allele frequency* | Conditional association with 25(OH)D (nmol/L) |
| --- | --- | --- | --- | --- | --- |
| 4:72617775 | rs1352846 | G | A | 0.29 | 0.172 |
| 4:72618334 | rs7041 | C | A | 0.43 | -0.045 |
| 4:72634343 | rs4694431 | T | C | 0.04 | -0.034 |
| 4:72770563 | rs139148694 | GTGCTTTTATCAA | G | na | 0.028 |
| 11:14339328 | rs16913816 | A | G | 0.03 | -0.031 |
| 11:14900931 | rs117913124 | A | G | 0.02 | 0.503 |
| 11:14912573 | rs117576073 | T | G | 0.02 | 0.246 |
| 11:14913575 | rs12794714 | A | G | 0.43 | 0.139 |
| 11:14913645 | rs202122669 | A | G | 0.001 | -0.615 |
| 11:14913900 | rs187639972 | C | G | 0.002 | -0.360 |
| 11:14941652 | rs117115472 | G | C | 0.001 | 0.148 |
| 11:71157867 | rs139168803 | A | G | 0.007 | -0.188 |
| 11:71158672 | rs12573951 | G | A | 0.06 | -0.045 |
| 11:71161063 | rs7928249 | G | A | 0.28 | -0.131 |
| 11:71180762 | rs549000212 | A | C | 0.001 | -0.364 |
| 11:71290740 | rs4081429 | C | A | 0.40 | 0.017 |
| 20:52714706 | rs6123359 | G | A | 0.11 | -0.026 |
| 20:52731402 | rs6127099 | T | A | 0.26 | 0.013 |
| 20:52735238 | rs35870583 | GT | G | 0.18 | 0.027 |
| 20:52737123 | rs2585442 | G | C | 0.28 | -0.025 |
| 20:52788925 | rs2762942 | A | G | 0.06 | -0.053 |

* obtained from non-Finnish European population (gnomad.broadinstitute.org)

# Supplementary Table 4. Non-linear associations between 25(OHD) and each outcome across five quantiles.

| **Outcome quantile** | **Mean 25(OH)D** | **minimum 25(OH)D** | **Maximum 25(OH)D** | **OR** | **95% CI** | | **p value** |
| --- | --- | --- | --- | --- | --- | --- | --- |
| All autoimmune diseases 1 | 35.2 | 27.4 | 47.7 | 0.958 | 0.838 | 1.094 | 0.525 |
| All autoimmune diseases 2 | 45.2 | 33.2 | 58.6 | 0.856 | 0.746 | 0.983 | 0.027 |
| All autoimmune diseases 3 | 54.1 | 40.5 | 69.0 | 1.078 | 0.936 | 1.241 | 0.298 |
| All autoimmune diseases 4 | 63.9 | 48.2 | 80.4 | 0.903 | 0.787 | 1.036 | 0.147 |
| All autoimmune diseases 5 | 78.4 | 58.4 | 91.3 | 0.983 | 0.859 | 1.125 | 0.801 |
| Autoimmunity subgroup 1 | 35.2 | 27.4 | 47.7 | 0.975 | 0.816 | 1.164 | 0.777 |
| Autoimmunity subgroup 2 | 45.2 | 33.2 | 58.6 | 0.875 | 0.727 | 1.053 | 0.156 |
| Autoimmunity subgroup 3 | 54.1 | 40.5 | 69.0 | 1.025 | 0.848 | 1.238 | 0.798 |
| Autoimmunity subgroup 4 | 63.9 | 48.2 | 80.4 | 1.051 | 0.874 | 1.264 | 0.597 |
| Autoimmunity subgroup 5 | 78.4 | 58.4 | 91.3 | 1.012 | 0.850 | 1.205 | 0.891 |
| Autoinflammation subgroup 1 | 35.2 | 27.4 | 47.7 | 0.948 | 0.787 | 1.141 | 0.573 |
| Autoinflammation subgroup 2 | 45.2 | 33.2 | 58.6 | 0.822 | 0.680 | 0.995 | 0.044 |
| Autoinflammation subgroup 3 | 54.1 | 40.5 | 69.0 | 1.128 | 0.926 | 1.374 | 0.231 |
| Autoinflammation subgroup 4 | 63.9 | 48.2 | 80.4 | 0.765 | 0.631 | 0.927 | 0.006 |
| Autoinflammation subgroup 5 | 78.4 | 58.4 | 91.3 | 0.918 | 0.756 | 1.116 | 0.391 |
| Ankylosing spondylitis 1 | 35.2 | 27.4 | 47.7 | 1.213 | 0.647 | 2.271 | 0.547 |
| Ankylosing spondylitis 2 | 45.2 | 33.2 | 58.6 | 0.932 | 0.517 | 1.681 | 0.816 |
| Ankylosing spondylitis 3 | 54.1 | 40.5 | 69.0 | 0.906 | 0.498 | 1.650 | 0.747 |
| Ankylosing spondylitis 4 | 63.9 | 48.2 | 80.4 | 0.778 | 0.427 | 1.416 | 0.411 |
| Ankylosing spondylitis 5 | 78.4 | 58.4 | 91.3 | 0.997 | 0.560 | 1.775 | 0.991 |
| Giant cell arteritis 1 | 35.2 | 27.4 | 47.7 | 1.144 | 0.402 | 3.250 | 0.801 |
| Giant cell arteritis 2 | 45.2 | 33.2 | 58.6 | 0.368 | 0.138 | 0.979 | 0.045 |
| Giant cell arteritis 3 | 54.1 | 40.5 | 69.0 | 0.808 | 0.321 | 2.036 | 0.651 |
| Giant cell arteritis 4 | 63.9 | 48.2 | 80.4 | 0.499 | 0.209 | 1.189 | 0.117 |
| Giant cell arteritis 5 | 78.4 | 58.4 | 91.3 | 0.674 | 0.279 | 1.628 | 0.381 |
| Graves’ disease 1 | 35.2 | 27.4 | 47.7 | 1.030 | 0.660 | 1.608 | 0.896 |
| Graves’ disease 2 | 45.2 | 33.2 | 58.6 | 0.945 | 0.603 | 1.481 | 0.805 |
| Graves’ disease 3 | 54.1 | 40.5 | 69.0 | 0.829 | 0.518 | 1.329 | 0.437 |
| Graves’ disease 4 | 63.9 | 48.2 | 80.4 | 1.098 | 0.671 | 1.795 | 0.710 |
| Graves’ disease 5 | 78.4 | 58.4 | 91.3 | 0.992 | 0.592 | 1.662 | 0.976 |
| Psoriatic arthritis 1 | 35.2 | 27.4 | 47.7 | 0.806 | 0.415 | 1.565 | 0.525 |
| Psoriatic arthritis 2 | 45.2 | 33.2 | 58.6 | 0.542 | 0.262 | 1.123 | 0.099 |
| Psoriatic arthritis 3 | 54.1 | 40.5 | 69.0 | 2.200 | 0.967 | 5.006 | 0.060 |
| Psoriatic arthritis 4 | 63.9 | 48.2 | 80.4 | 0.731 | 0.336 | 1.594 | 0.431 |
| Psoriatic arthritis 5 | 78.4 | 58.4 | 91.3 | 0.750 | 0.341 | 1.650 | 0.474 |
| Polymyalgia rheumatica 1 | 35.2 | 27.4 | 47.7 | 0.932 | 0.554 | 1.568 | 0.792 |
| Polymyalgia rheumatica 2 | 45.2 | 33.2 | 58.6 | 1.803 | 1.059 | 3.068 | 0.030 |
| Polymyalgia rheumatica 3 | 54.1 | 40.5 | 69.0 | 1.175 | 0.722 | 1.912 | 0.518 |
| Polymyalgia rheumatica 4 | 63.9 | 48.2 | 80.4 | 1.263 | 0.799 | 1.995 | 0.317 |
| Polymyalgia rheumatica 5 | 78.4 | 58.4 | 91.3 | 1.164 | 0.752 | 1.801 | 0.496 |
| Rheumatoid arthritis 1 | 35.2 | 27.4 | 47.7 | 0.969 | 0.745 | 1.260 | 0.815 |
| Rheumatoid arthritis 2 | 45.2 | 33.2 | 58.6 | 0.740 | 0.559 | 0.979 | 0.035 |
| Rheumatoid arthritis 3 | 54.1 | 40.5 | 69.0 | 1.060 | 0.787 | 1.426 | 0.703 |
| Rheumatoid arthritis 4 | 63.9 | 48.2 | 80.4 | 1.058 | 0.793 | 1.410 | 0.702 |
| Rheumatoid arthritis 5 | 78.4 | 58.4 | 91.3 | 0.920 | 0.699 | 1.212 | 0.553 |
| Sjögren's syndrome 1 | 35.2 | 27.4 | 47.7 | 0.997 | 0.388 | 2.561 | 0.995 |
| Sjögren's syndrome 2 | 45.2 | 33.2 | 58.6 | 0.525 | 0.171 | 1.607 | 0.259 |
| Sjögren's syndrome 3 | 54.1 | 40.5 | 69.0 | 2.074 | 0.601 | 7.155 | 0.248 |
| Sjögren's syndrome 4 | 63.9 | 48.2 | 80.4 | 0.790 | 0.225 | 2.770 | 0.713 |
| Sjögren's syndrome 5 | 78.4 | 58.4 | 91.3 | 1.020 | 0.370 | 2.811 | 0.970 |
| Systemic lupus erythematosus 1 | 35.2 | 27.4 | 47.7 | 0.706 | 0.328 | 1.519 | 0.373 |
| Systemic lupus erythematosus 2 | 45.2 | 33.2 | 58.6 | 0.467 | 0.203 | 1.077 | 0.074 |
| Systemic lupus erythematosus 3 | 54.1 | 40.5 | 69.0 | 0.486 | 0.193 | 1.223 | 0.125 |
| Systemic lupus erythematosus 4 | 63.9 | 48.2 | 80.4 | 0.832 | 0.331 | 2.093 | 0.696 |
| Systemic lupus erythematosus 5 | 78.4 | 58.4 | 91.3 | 1.339 | 0.570 | 3.148 | 0.503 |
| Systemic sclerosis 1 | 35.2 | 27.4 | 47.7 | 1.763 | 0.500 | 6.222 | 0.378 |
| Systemic sclerosis 2 | 45.2 | 33.2 | 58.6 | 1.504 | 0.324 | 6.978 | 0.602 |
| Systemic sclerosis 3 | 54.1 | 40.5 | 69.0 | 2.028 | 0.529 | 7.779 | 0.303 |
| Systemic sclerosis 4 | 63.9 | 48.2 | 80.4 | 1.361 | 0.224 | 8.256 | 0.738 |
| Systemic sclerosis 5 | 78.4 | 58.4 | 91.3 | 0.362 | 0.091 | 1.438 | 0.149 |
| Autoimmune hepatitis 1 | 35.2 | 27.4 | 47.7 | 0.521 | 0.149 | 1.825 | 0.308 |
| Autoimmune hepatitis 2 | 45.2 | 33.2 | 58.6 | 3.382 | 0.491 | 23.277 | 0.216 |
| Autoimmune hepatitis 3 | 54.1 | 40.5 | 69.0 | 1.100 | 0.132 | 9.178 | 0.930 |
| Autoimmune hepatitis 4 | 63.9 | 48.2 | 80.4 | 0.186 | 0.041 | 0.847 | 0.030 |
| Autoimmune hepatitis 5 | 78.4 | 58.4 | 91.3 | 3.720 | 0.732 | 18.910 | 0.113 |
| Crohn’s disease 1 | 35.2 | 27.4 | 47.7 | 0.925 | 0.590 | 1.449 | 0.733 |
| Crohn’s disease 2 | 45.2 | 33.2 | 58.6 | 0.863 | 0.545 | 1.368 | 0.531 |
| Crohn’s disease 3 | 54.1 | 40.5 | 69.0 | 1.795 | 1.077 | 2.990 | 0.025 |
| Crohn’s disease 4 | 63.9 | 48.2 | 80.4 | 0.760 | 0.475 | 1.215 | 0.251 |
| Crohn’s disease 5 | 78.4 | 58.4 | 91.3 | 0.815 | 0.503 | 1.320 | 0.405 |
| Coeliac disease 1 | 35.2 | 27.4 | 47.7 | 1.249 | 0.790 | 1.976 | 0.341 |
| Coeliac disease 2 | 45.2 | 33.2 | 58.6 | 0.843 | 0.533 | 1.334 | 0.467 |
| Coeliac disease 3 | 54.1 | 40.5 | 69.0 | 1.243 | 0.787 | 1.961 | 0.351 |
| Coeliac disease 4 | 63.9 | 48.2 | 80.4 | 0.863 | 0.567 | 1.311 | 0.489 |
| Coeliac disease 5 | 78.4 | 58.4 | 91.3 | 1.108 | 0.773 | 1.588 | 0.575 |
| Hashimoto’s thyroiditis 1 | 35.2 | 27.4 | 47.7 | 1.060 | 0.267 | 4.213 | 0.934 |
| Hashimoto’s thyroiditis 2 | 45.2 | 33.2 | 58.6 | 2.121 | 0.489 | 9.199 | 0.315 |
| Hashimoto’s thyroiditis 3 | 54.1 | 40.5 | 69.0 | 1.083 | 0.281 | 4.172 | 0.908 |
| Hashimoto’s thyroiditis 4 | 63.9 | 48.2 | 80.4 | 1.164 | 0.224 | 6.064 | 0.857 |
| Hashimoto’s thyroiditis 5 | 78.4 | 58.4 | 91.3 | 0.271 | 0.070 | 1.053 | 0.059 |
| Primary biliary cholangitis 1 | 35.2 | 27.4 | 47.7 | 1.205 | 0.375 | 3.871 | 0.754 |
| Primary biliary cholangitis 2 | 45.2 | 33.2 | 58.6 | 0.391 | 0.091 | 1.678 | 0.206 |
| Primary biliary cholangitis 3 | 54.1 | 40.5 | 69.0 | 1.505 | 0.380 | 5.967 | 0.561 |
| Primary biliary cholangitis 4 | 63.9 | 48.2 | 80.4 | 1.930 | 0.484 | 7.696 | 0.352 |
| Primary biliary cholangitis 5 | 78.4 | 58.4 | 91.3 | 2.120 | 0.636 | 7.068 | 0.221 |
| Psoriasis 1 | 35.2 | 27.4 | 47.7 | 0.884 | 0.680 | 1.148 | 0.356 |
| Psoriasis 2 | 45.2 | 33.2 | 58.6 | 0.772 | 0.586 | 1.019 | 0.067 |
| Psoriasis 3 | 54.1 | 40.5 | 69.0 | 0.950 | 0.713 | 1.264 | 0.723 |
| Psoriasis 4 | 63.9 | 48.2 | 80.4 | 0.804 | 0.602 | 1.074 | 0.140 |
| Psoriasis 5 | 78.4 | 58.4 | 91.3 | 0.777 | 0.584 | 1.034 | 0.083 |
| Type 1 diabetes mellitus 1 | 35.2 | 27.4 | 47.7 | 0.672 | 0.252 | 1.794 | 0.428 |
| Type 1 diabetes mellitus 2 | 45.2 | 33.2 | 58.6 | 1.373 | 0.485 | 3.887 | 0.550 |
| Type 1 diabetes mellitus 3 | 54.1 | 40.5 | 69.0 | 0.846 | 0.283 | 2.529 | 0.765 |
| Type 1 diabetes mellitus 4 | 63.9 | 48.2 | 80.4 | 1.786 | 0.553 | 5.770 | 0.332 |
| Type 1 diabetes mellitus 5 | 78.4 | 58.4 | 91.3 | 1.745 | 0.506 | 6.021 | 0.378 |
| Ulcerative colitis 1 | 35.2 | 27.4 | 47.7 | 0.803 | 0.570 | 1.133 | 0.212 |
| Ulcerative colitis 2 | 45.2 | 33.2 | 58.6 | 0.902 | 0.640 | 1.272 | 0.557 |
| Ulcerative colitis 3 | 54.1 | 40.5 | 69.0 | 1.099 | 0.777 | 1.554 | 0.593 |
| Ulcerative colitis 4 | 63.9 | 48.2 | 80.4 | 0.739 | 0.532 | 1.025 | 0.070 |
| Ulcerative colitis 5 | 78.4 | 58.4 | 91.3 | 1.223 | 0.865 | 1.727 | 0.254 |

# Supplementary Table 5. Two-sample MR pleiotropy robust sensitivity analyses.

| **Outcome** | **Method** | **No. SNPs** | **OR*** | **95% CI** | | **p value** |
| --- | --- | --- | --- | --- | --- | --- |
| Psoriasis | Inverse variance weighted | 2 | 0.518 | 0.278 | 0.963 | 0.038 |
| Systemic lupus erythematosus | Inverse variance weighted | 12 | 0.610 | 0.401 | 0.928 | 0.021 |
| Systemic lupus erythematosus | MR Egger | 12 | 0.506 | 0.220 | 1.163 | 0.140 |
| Systemic lupus erythematosus | Weighted median | 12 | 0.613 | 0.340 | 1.107 | 0.104 |
| Systemic lupus erythematosus | Weighted mode | 12 | 0.616 | 0.339 | 1.118 | 0.139 |
| Giant cell arteritis | Inverse variance weighted | 14 | 0.948 | 0.474 | 1.899 | 0.881 |
| Giant cell arteritis | MR Egger | 14 | 0.747 | 0.135 | 4.138 | 0.744 |
| Giant cell arteritis | Weighted median | 14 | 1.157 | 0.522 | 2.562 | 0.719 |
| Giant cell arteritis | Weighted mode | 14 | 1.213 | 0.539 | 2.732 | 0.648 |
| Polymyalgia rheumatica | Inverse variance weighted | 14 | 0.975 | 0.715 | 1.328 | 0.871 |
| Polymyalgia rheumatica | MR Egger | 14 | 0.755 | 0.362 | 1.575 | 0.468 |
| Polymyalgia rheumatica | Weighted median | 14 | 0.951 | 0.635 | 1.422 | 0.805 |
| Polymyalgia rheumatica | Weighted mode | 14 | 1.025 | 0.706 | 1.489 | 0.897 |

*Estimates scaled to per 10 nmol/L increase in 25(OH)D
